# The Muscles of the Fore-arm

**Published:** 1890-11

**Authors:** A. L. Benedict

**Affiliations:** Buffalo, N. Y.


### THE MUSCLES OF THE FORE-ARM.

By A. L. BENEDICT, M. D., Buffalo, N. Y.

The accompanying table and generalizations concerning the muscles of the fore-arm are the result of anatomical studies, both before and since graduation. Several of my students have found them a help, and, on that account, I offer them to others with the hope that they may serve to render less chaotic the mental picture of this important region. No originality is claimed, except for some phases of the presentation of the subject; nor is it expected that all the difficulties attending the study of the anatomy of these muscles will be removed.

Of the nineteen muscles of the fore-arm—two pronate the radius; two supinate the radius; three flex the hand; three extend the hand; three flex the fingers and thumb; three extend the fingers; three extend the thumb; — eight anterior; eleven posterior.

Both the anterior and the posterior group may be divided into superficial and deep layers. At the upper part of the fore-arm, the flexor sublimis forms an intermediate layer.

The course of the superficial muscles may be represented by lines radiating from the internal and the external condyle of the humerus along the front and the back of the fore-arm, respectively

With the exception of the supinator longus and the extensor carpi radialis longior, the superficial muscles arise, at least in part, from a condyle by a common tendon.

One supinator and one pronator are superficial and lead the lists of superficial muscles, one supinator and one pronator are deep and have no analogy with other deep muscles. With the necessary exceptions of the pronator quadratus and the supinator brevis, all deep muscles arise from shaft and interosseous membrane.

At the inner (ulnar) side of the fore-arm, the anterior and posterior groups are separated by the attachment of both the extensor and the flexor carpi ulnaris to the posterior border of the ulna. At the outer (radial side), there is no marked separation, and the supinator longus over-laps nearly half of the front of the fore-arm.

*Insertion.*—The pronators and supinators, of course, are inserted into the radius. The flexors and extensors of the hand are inserted into the bases of metacarpal bones, except the palmaris longus. The flexor carpi ulnaris is also attached to the pisiform bone.

The extensors of the fingers are inserted by middle slips into the bases of the second phalanges, and by lateral slips, which unite, into the bases of the distal phalanges.

The flexor *profundus* is the flexor *perforans*, and it is inserted into the *end phalanges*. Similarly, the flexor longus pollicis is inserted into the distal phalanx of the thumb. This is in accordance with a general rule that a "long" muscle is long at both ends, *i. e.*, it arises higher and it is inserted lower than the corresponding "short" muscle.

The extensor indices and the extensor minimi digiti are inserted in common with the corresponding tendons of the extensor communis digitorum.

The extensors of the thumb are inserted into the bases of the bones from which they are named. The extensor ossis metacarpi, which is inserted into the largest bone, has the largest origin, *i. e.*, from both bones of the fore-arm. The extensor primi internodii, whose tendon runs with that of the extensor ossis metacarpi, on the radial side of the "anatomical snuff box," arises from the radius. The extensor secundi internodii, whose tendon forms the ulnar side of the "snuff box," arises from the ulna.

The proximal phalanges of the fingers have no muscular insertions, except from the small muscles of the hand, and, therefore, they are flexed and extended only by the muscles which are inserted into the second and third phalanges. Hence, the rule to amputate the entire finger if the base of the second phalanx cannot be saved. An exception is sometimes made in the case of the index.

### MUSCLES OF THE FORE-ARM.

### Anterior Group.

| Muscle | Layer | Origin. | | | Insertion. |
|---|---|---|---|---|---|
| Pronator Radii Teres. | Superficial. | (1) Internal Condyle by common tendon. Fascia and Inter-muscular septa. | (2) Inner side of Coronoid. | | Radius. Rough ridge on middle of outer [border. |
| Flexor Carpi Radialis. | Superficial. | (1) Internal Condyle by common tendon. Fascia and Inter-muscular septa. | | | Metacarpal. Base of 2d. |
| Palmaris Longus. | Superficial. | (1) Internal Condyle by common tendon. Fascia and Inter-muscular septa. | | | Palmar fascia. |
| Flexor Carpi Ulnaris. | Superficial. | (1) Internal Condyle by common tendon. Fascia and Inter-muscular septa. | | (2) Upper $\frac{2}{3}$ post border of ulna and septum. | Metacarpal. Base of 5th and Pisiform. |
| Flexor Sublimis Dig. | Middle | (1) Internal Condyle by common tendon. Fascia and Inter-muscular septa. | (2) “ | (3) Oblique line of radius. | 2d. Phalanges of 4 fingers. |
| Flexor Profundus Dig. | Deep. | | “ | Upper $\frac{2}{3}$ shaft of ulna and interos mem. | 3d. “ “ “ |
| Flexor Longus Pol. | Deep. | | | “ “ “ “ radius “ “ “ | 2d. Phalanx of thumb. |
| Pronator Radii Quad. | Deep. | | | Lower $\frac{1}{4}$ of ant. border of ulna. | Radius. Lower $\frac{1}{4}$ of outer border. |

### Posterior Group.

| Muscle | Layer | Origin | | | Insertion. |
|---|---|---|---|---|---|
| Supinator Radii Longus. | Superficial. | Upper $\frac{2}{3}$ Ext. condyloid ridge | | and intermusc. septum | Radius. Base of styloid process. |
| Ext. Carpi Rad. Long. | Superficial. | Lower $\frac{1}{3}$ “ “ “ | | “ “ “ | Metacarpal. Base of 2d. |
| “ “ “ Brev. | Superficial. | External Condyle by Common Tendon | Ext. lat. lig. Deep fascia. | “ “ “ | “ “ 3d. |
| Ext. Communis Dig. | Superficial. | External Condyle by Common Tendon | Ext. lat. lig. Deep fascia. | “ “ “ | 2d and 3d Phalanges of 4 fingers. |
| Ext. Minimi Dig. | Superficial. | External Condyle by Common Tendon | Ext. lat. lig. Deep fascia. | “ “ “ | “ “ “ “ little finger. |
| Ext. Carpi Ulnaris. | Superficial. | External Condyle by Common Tendon | “ “ | “ “ “ | Metacarpal. Base of 5th. |
| Supinator Radii Brev. | | Ext. condyle, Ext. lat. and orbic ligaments, oblique line of ulna. | | | Radius. Inner surf. of neck, outer surf. of bicip. tuberosity, oblique [line. |
| Ext. Ossis Metac. Pol. | Deep. | Posterior surface of shaft of radius and ulna | | and interos. mem. | Base of Metacarpal of thumb. |
| “ Primi Intern Pol. | Deep. | “ “ “ radius | | “ “ “ | “ “ 1st Phalanx “ “ |
| “ Secundi Intern Pol. | Deep. | “ “ “ ulna | | “ “ “ | “ “ 2d “ “ “ |
| “ Indicis. | Deep. | “ “ “ ulna | | “ “ “ | 2d and 3d Phalanges of index. |

